# ALAT-SEPAR Consensus on the Definition and Classification of Asthma Exacerbations by Severity: A Move Toward International Standardization^☆^

**DOI:** 10.1016/j.opresp.2025.100469

**Published:** 2025-11-05

**Authors:** Ana María Stok, Francisco Álvarez-Gutiérrez, Lilian S. Ballini Caetano, Marina Blanco-Aparicio, Francisco Casas-Maldonado, Carmen Cano, Patricia Fernández, Gabriel García, Alicia Padilla-Galo, Vicente Plaza, Ignacio Zabert, José Gregorio Soto-Campos

**Affiliations:** a Instituto de Investigaciones en Patologías Respiratorias, San Miguel de Tucumán, Tucumán, Argentina; b Hospital Universitario Virgen del Rocío, Sevilla, Spain; c División de Neumología, Universidad Federal de Sao Paulo, UNIFESP-EPM, Brazil; d Hospital Universitario de A Coruña, Spain; e Hospital Universitario Clínico San Cecilio, Granada, Spain; f Clínica de Asma, Instituto Nacional de Enfermedades Respiratorias Ismael Cosio Villegas, Ciudad de México, Mexico; g CIMER Centro de Investigación en Medicina Respiratoria, Chile; h Centro en Investigaciones Respiratoria de La Plata, CEPIR, La Plata, Argentina; i Hospital Universitario Virgen de la Victoria, Málaga, Spain; j Hospital de la Santa Creu i Sant Pau, Barcelona, Spain; k Fisiología Humana, Facultad de Ciencias Médicas, Universidad Nacional del Comahue, Argentina; l Hospital universitario de Jerez, Cádiz, Spain

**Keywords:** Asthma, Consensus, Exacerbation, Definition, Asthma management, Asma, Consenso, Exacerbación, Definición, Tratamiento del asma

## Abstract

Asthma exacerbation is a significant clinical event that occurs with varying severity, yet a set of universally accepted standardized definitions is still needed. The aim of this consensus developed by ALAT and SEPAR was to fill this gap with a validated proposal for classifying asthma exacerbations into levels of severity: non-severe, severe, and very severe.

The consensus was based on an in-depth review of the literature conducted by the scientific committee to identify key parameters for each level of severity, including worsening symptoms, respiratory function changes, and the need for specific medical interventions. A total of 67 publications were analyzed to generate a questionnaire on the defining elements of asthma exacerbations, and this was put to the vote. Twenty-eight international experts participated in the validation of characteristics and definitions of exacerbation severity following Delphi methodology. The resulting definitions clearly distinguish non-severe exacerbations (requiring minor adjustments in treatment) from severe exacerbations (requiring more intensive interventions, including longer systemic corticosteroid use or hospitalization) and very severe exacerbations (life-threatening events requiring intensive care). These definitions provide a standardized framework that facilitates comparison between clinical trials and optimizes patient care.

This consensus lays the foundation for unifying management criteria in global clinical practice and fostering research on the efficacy of asthma treatments. It also underlines the importance of accurate classification in improving clinical outcomes and reducing the overall burden of disease.

## Abstract (es)

La exacerbación de asma es un acontecimiento clínico significativo que varía en gravedad y carece de definiciones estandarizadas universalmente aceptadas. Este consenso, desarrollado por la Asociación Latinoamericana de Tórax (ALAT) y la Sociedad Española de Neumología y Cirugía Torácica (SEPAR), busca llenar este vacío mediante una propuesta validada para clasificar las exacerbaciones asmáticas en niveles de gravedad: no graves, graves y muy graves.

El consenso se basó en una revisión exhaustiva de la literatura realizada por el Comité Científico, que identificó parámetros clave para cada nivel de gravedad, como el deterioro de los síntomas, alteraciones funcionales respiratorias y la necesidad de intervenciones médicas específicas. En total, se analizaron 67 publicaciones para generar un cuestionario sobre elementos definitorios de las exacerbaciones de asma que fue sometido a votación. Veintiocho expertos internacionales participaron en la validación de características y de definiciones de gravedad de exacerbación siguiendo metodología Delphi. Las definiciones resultantes distinguen claramente las exacerbaciones no graves, que requieren ajustes menores en el tratamiento, de las graves, que demandan intervenciones más intensivas, como el uso más prolongado de corticoesteroides sistémicos u hospitalización, y de las muy graves, que implican un riesgo vital y necesitan cuidados intensivos. Estas definiciones proporcionan un marco homogéneo que facilita la comparación entre estudios clínicos y optimiza la atención al paciente.

Este consenso establece las bases para unificar criterios de manejo en la práctica clínica global y promover la investigación sobre la eficacia de tratamientos para el asma. Además, subraya la importancia de una clasificación precisa para mejorar los resultados clínicos y reducir la carga global de la enfermedad.

## Introduction

Asthma affects more than 300 million people globally and is one of the most common chronic respiratory diseases worldwide.^1^ Prevalence in Spain is around 5% in adults and 10% in children.^2^, ^3^ In Latin America, prevalence is estimated to be around 7% in adults, while in the pediatric population it can be as high as 17%. Country-specific rates vary widely, ranging from 5% in some Mexican cities to 30% in Costa Rica.^2^, ^4^, ^5^ Asthma continues to be a major health challenge and economic burden, with an estimated mortality of 461,000 individuals in 2019. In Mexico, the average annual cost per patient to the public health system is 43,813.92 Mexican pesos (∼2070 EUR/2170 USD), compared to 1533 EUR (∼1610 USD) in Spain.^6^, ^7^, ^8^ These data underline the importance of effective management to improve patient quality of life and reduce the overall disease burden.

The definition of asthma severity is well established in the medical literature and supported by various international and national guidelines, such as the Global Initiative for Asthma (GINA) guidelines and the Spanish Asthma Management Guidelines (GEMA).^9^, ^10^ These guidelines provide a detailed framework for classifying asthma according to frequency and symptom control. However, there is no standard definition for the severity of asthma exacerbation and considerable variability is observed across the scientific literature. In 2009, the American Thoracic Society (ATS) and the European Respiratory Society (ERS) created a joint task force to address various aspects and definitions of asthma control and exacerbations and make a series of recommendations, especially in the context of clinical trials; their work highlighted the need for greater standardization.^11^ Several variables are currently used to classify exacerbations in clinical practice and studies, including frequency, annualized rate, and specific clinical parameters, such as forced expiratory volume in 1 second (FEV_1_), peak expiratory flow (PEF), respiratory rate, and oxygen saturation.^12^, ^13^ In 2019, the ERS and the European Academy of Allergy and Clinical Immunology (EAACI) defined severe exacerbation as an event in which the patient requires 3 or more days of systemic corticosteroids, is treated in the emergency department, or is hospitalized,^14^ referring to the ERS/ATS Guidelines.^11^

The current lack of a comprehensive, universally accepted definition of asthma exacerbation according to severity has led to an unacceptable heterogeneity in the way these events are documented and managed in different contexts.^15^ Although the ERS/ATS Guidelines definition of severe exacerbation has been widely used in clinical trials since its publication,^11^ the lack of a standardized, comprehensive classification of exacerbations limits the comparability of clinical trial results and may affect the quality of patient care. A combination of certain criteria, such as the percentage of predicted FEV_1_, respiratory rate, and the need for medical intervention, could label an exacerbation as moderate or severe, depending on the classification used. The definitions of exacerbation encompass events that range from moderate to severe or potentially fatal.^16^, ^17^

Standardizing these definitions according to severity is therefore crucial to improve the accuracy and comparability of data in clinical trials, optimize patient care in clinical practice, facilitate the transfer of research results to clinical practice, and promote better asthma management globally. To this end, this consensus statement seeks to address this knowledge gap with a proposal validated by an international panel of asthma specialists.

## Material and methods

### Study design and scientific committee

This is an observational consensus study based on the Delphi methodology. A scientific committee (SC) of 12 professionals representing SEPAR and ALAT in equal numbers was established. The SC was involved in all the project processes but did not participate in the Delphi panel in order to avoid bias in the results.

An exhaustive literature search was performed using the PubMed database and the Virtual Health Library (VHL) to define the items to be included in the definitions of asthma. The search was restricted to terms that appeared in the title or abstract, and general search terms (not MeSH) were used to include all articles, not only those indexed in MEDLINE (Supplementary Table 1). The selected papers included some definition for asthma exacerbation of any severity (Supplementary Fig. 1, Supplementary Table 2). The definitions and their components were extracted and itemized for individual evaluation and categorized according to the defined severity as: very severe exacerbation (life-threatening), severe exacerbation, and non-severe exacerbation.

### Survey questionnaires and Delphi procedure

In order to reflect as far as possible, the procedures and practices of standard clinical practice, the SC's first step was to design an initial survey, based on its evaluation of the results of the literature search (Supplementary Table 3). In this survey, the panel gave their opinion on whether the items selected as possible characteristics associated with each level of exacerbation should be included in the corresponding definition. Questions were designed to obtain a yes/no answer, as follows: “Do you think [item] defines a very severe/severe/non-severe exacerbation?”

The SC used the results to draw up definitions and additional proposals that were submitted for consensus using the modified RAND/UCLA Delphi panel methodology.^18^ According to this procedure, participants were asked to rate their agreement or disagreement with each statement on the definitions of exacerbation on a 9-point Likert scale (from 1 = strongly disagree to 9 = strongly agree).

Survey questionnaires (1 round) and the Delphi consensus (2 rounds) were distributed to invited panelists from the participating societies via an interactive online platform and answered anonymously. The first survey and the 2 Delphi rounds were conducted in the first fortnights of April, June and July of 2024, respectively.

The SC evaluated the results in 2 interim meetings held after the initial survey and after the first round of the Delphi questionnaire, respectively. In the first interim meeting, the SC took into account the comments of the expert panel and modified and selected the round 1 Delphi questionnaire statements accordingly. In the second meeting, they passed those that did not reach consensus in the first round to the second round.

### Panelists

The experts invited to participate in the panel were members of the scientific societies SEPAR and ALAT (Supplementary Table 4). The inclusion criteria were: 1) member of either scientific society mentioned, and 2) relevant experience in the management of patients with severe asthma, with a minimum of 5 years practice in the field.

### Consensus definition and statistical analysis

The Delphi consensus was based on the Rand Healthcare Corporation and the University of California Los Angeles (Rand/UCLA) appropriateness method.^18^

Statements were classified as inappropriate, uncertain, or appropriate when a median score of 1–3, 4–6, or 7–9, respectively, was recorded. Consensus was reached when at least two thirds of the individual scores fell within the median range; otherwise, it was classified as no consensus. However, the statement was undecided when more than one third of the individual scores fell in the range that did not contain the median. All analyses were performed using R statistical software version 3.2.5.

## Results

### Literature review

After duplicate screening and exclusion of publications that did not contain an explicit definition of asthma exacerbation, 67 publications were included for the analysis of definitions. Late-stage clinical studies of an intervention were excluded when an early-stage study of that same intervention had already been included (Supplementary Fig. 1).

### Development of the questionnaire and participants

The SC analyzed the compiled literature in a working meeting and designed the initial series of proposed definitions for asthma exacerbation (Supplementary Table 2). Thirty-one experts were invited to vote on the proposals, of whom 28 responded to the initial survey (Supplementary Table 3).

### Consensus results

#### Components of the definition of very severe (life-threatening) asthma exacerbation

All components of the definition of very severe exacerbation that involves a risk to life reached consensus in the first round (Table 1).

**Table 1:** Characteristics associated with very severe asthma exacerbation (life-threatening) agreed by the expert panel.

Statements that reached consensus in the first round		Median (range 1–9)
1. Cursa con paro respiratorio.	Respiratory arrest involved.	8.5
2. Precisa de ventilación mecánica.	Event requires mechanical ventilation.	9
3. Cursa con hipercapnia con PaCO_2_ > 45 mm Hg siempre y cuando el paciente no sufra una situación de insuficiencia respiratoria hipercápnica previa.	Event presents with hypercapnia with a PaCO_2_ > 45 mm Hg as long as the patient is not suffering from a previous situation of hypercapnic respiratory failure.	9
4. Cursa con acidosis respiratoria (pH < 7.30).	Event leads to respiratory acidosis (pH < 7.30).	8.5
5. Cursa con movimiento paradójico abdominal.	Paradoxical abdominal movement present.	9
6. Cursa con deterioro del nivel de conciencia ocasionada por la exacerbación.	Decline in the level of consciousness is caused by exacerbation.	9
7. Cursa con inestabilidad hemodinámica.	Hemodynamic instability involved.	8.5
8. Requiere atención en una unidad de cuidados intensivos (UCI) o unidad de cuidados intermedios respiratorios (UCRI).	The event requires attention in an intensive care unit (ICU) or an intermediate respiratory care unit (IRCU).	9

#### Components of the definition of severe asthma exacerbation

In the first round, there was consensus among panelists that 12 components of the definition of severe exacerbation were appropriate (Table 2). A 13th element was withdrawn after the first round (patients with severe exacerbation should have a PaCO_2_ > 45 mm Hg). As this statement reached consensus as a definition of very severe exacerbation (Table 1), the SC agreed that it did not need to be submitted to a second round of voting for this definition.

**Table 2:** Characteristics associated with severe asthma exacerbation agreed by the expert panel.

Statements that reached consensus in the first round		Median (range 1–9)
1. Presenta una frecuencia respiratoria > 30 rpm.	Respiratory rate > 30 breaths per minute.	9
2. Presenta frecuencia cardiaca > 120 lpm.	Heart rate > 120 beats per minute.	8.5
3. Presenta FEV_1_ o PEF < 50% del valor de referencia personal previo o del mejor valor previo registrado.	FEV_1_ or PEF < 50% of the patient's previous reference value or the best previously recorded value.	9
4. Presenta SaO_2_ < 90% (<92% en pacientes embarazadas).	SpO_2_ < 90% (<92% in pregnant patients).	8.5
5. Presenta PaO_2_ < 60 mm Hg.	PaO_2_ < 60 mmHg.	8
6. Presenta insuficiencia respiratoria no hipercápnica.	Non-hypercapnic respiratory failure.	8
7. El paciente habla con palabras sueltas.	The patient speaks in single words.	8
8. El paciente presenta respiración con músculos accesorios.	The patient breathes with accessory muscles.	8
9. Requiere ingreso hospitalario.	Hospital admission required.	9
10. Requiere atención en un servicio de urgencias (≥24 h).	Emergency department care needed for <24 h.	9
11. Requiere de un ciclo de corticoesteroides sistémicos durante ≥5 días para controlar la exacerbación.	Course of systemic corticosteroids for ≥5 days is needed to control the exacerbation.	8.5
12. En pacientes que reciben un tratamiento de mantenimiento con corticoesteroides orales, requiere un incremento del tratamiento (hasta el doble de la dosis habitual) durante ≥3 días controlar la exacerbación.	Patients receiving maintenance treatment with oral corticosteroids require an escalation (up to double the stable maintenance dosage) of treatment for ≥3 days to control the exacerbation.	8.5

#### Components of the definition of non-severe asthma exacerbation

Most of the components (11/15) of the definition of non-severe asthma exacerbation reached consensus in the first round of the Delphi questionnaire. The 4 components that did not reach consensus were related to treatment. Of these, 1 was withdrawn, and the other 3 were revised and re-written for the second round. In the second round, 2 of these components reached consensus (Table 3).

**Table 3:** Characteristics associated with non-severe asthma exacerbation agreed by the expert panel.

Statements that reached consensus in the first round		Median (range 1–9)
Aumento de los síntomas asmáticos (p.ej., tos, sibilancias, opresión en el pecho y disnea).	Increased asthma symptoms (e.g., cough, wheezing, chest tightness, and shortness of breath).	9
Presenta una frecuencia del pulso 100–120 lpm.	Heart rate 100–120 beats per minute.	8
Presenta una SaO_2_ > 90% (>92% en pacientes embarazadas).	SpO_2_ > 90% (>92% in pregnant patients).	8
Presenta FEV_1_ o PEF del > 50% del valor de referencia personal previo/	FEV_1_ or PEF is >50% of the patient's previous reference value.	8
Presenta un aumento de la frecuencia respiratoria <25 rpm.	Respiratory rate increased < 25 bpm.	8
El paciente puede hablar con frases.	The patient can speak full sentences.	8
Requiere atención en un servicio de urgencias durante <24 horas	Emergency department care needed for <24 h.	8
No precisa de ingreso hospitalario.	Hospital admission not required.	9
Requiere un tratamiento adicional para prevenir la progresión a una exacerbación grave.	Additional treatment required to prevent progression to severe exacerbation.	9
No requiere tratamiento con corticoesteroides sistémicos por vía parenteral.	Treatment with parenterally administered systemic corticosteroids not required	8
Requiere el uso de corticoesteroides sistémicos >3 días si está contemplado en el plan de automanejo del paciente.	Systemic corticosteroids for >3 days needed, if included in the patient's self-management plan.	8

Statements that reached consensus in the second round		
Requiere tratamiento con corticoesteroides sistémicos durante <5 días para controlar la exacerbación.	Treatment with systemic corticosteroids for <5 days needed to control the exacerbation.	8
En pacientes que reciben un tratamiento de mantenimiento con corticoesteroides orales, requiere un incremento del tratamiento (hasta el doble de la dosis habitual) durante <3 días para controlar la exacerbación.	In patients receiving maintenance treatment with oral corticosteroids, escalation (up to double the stable maintenance dose) of treatment for <3 days is needed to control the exacerbation.	8

Statements that did not reach consensus or a median score		
No requiere tratamiento con corticoesteroides sistémicos.	Treatment with systemic corticosteroids not required.	5^a^
Requiere un incremento temporal del tratamiento con corticoesteroides orales de mantenimiento.	Temporary increase in background oral corticosteroid dosage required.	7.5^b^

a Remained inconclusive in both rounds.

b Withdrawn after the first round on the decision of the SC, as it was considered inconclusive.

## Discussion

This collaborative effort on the part of the scientific societies ALAT and SEPAR responds to the need for a clear, standardized definition of exacerbations voiced by a large majority of health professionals who treat patients with asthma. The first step taken was to analyze a range of classifications used in clinical trials, studies and secondary literature in order to pool evidence and experience in a validated quantitative consensus methodology adapted to the needs of this project.

### Definition of exacerbation in asthma

The prevailing lack of standard definitions is largely due to the complexity inherent to the classification of asthma exacerbations. Clearly, general exacerbation is commonly defined as a loss of asthma control that may result in the use of systemic corticosteroids, an increase in the dose of oral corticosteroids for 3 days or more, or a specific hospitalization,^19^ but there are no unified criteria to unequivocally define these events.^20^, ^21^, ^22^, ^23^ In medical literature, the criteria are unclear in terms of type and length of hospitalization and duration of treatment, so the definitions are insufficiently robust to deal with the wide range of cases encountered in routine practice. Variability in the interpretation of these severity levels can lead to confusion in clinical practice and an underestimation of the impact of these exacerbations on patient quality of life. The lack of a clear consensus on these definitions makes them inconclusive and difficult to apply in the development and analysis of new clinical studies and in routine clinical practice.

**Fig. 1:**
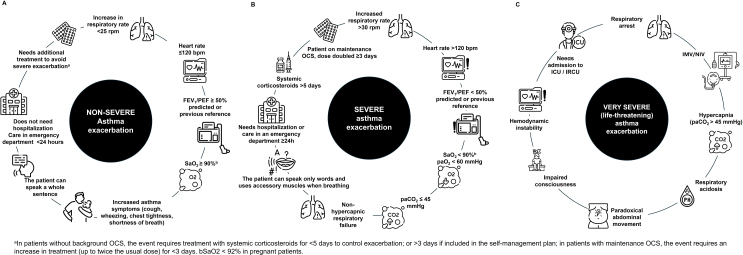
Consensus definitions for asthma exacerbations according to severity (A) Non-severe asthma exacerbation; (B) Severe asthma exacerbation; (C) Very severe asthma exacerbation. FEV_1_: forced expiratory volume in 1 second; bpm: beats per minute; PAO_2_: partial oxygen pressure; PaCO_2_: partial pressure of carbon dioxide; PEF: peak expiratory flow; rpm: respirations per minute; SaO_2_: oxygen saturation; ICU: intensive care unit; IRCU: intermediate respiratory care unit; IMV: invasive mechanical ventilation; NIMV: non-invasive mechanical ventilation.

The definition of severe exacerbation proposed by the ARS/ETS Guideline^11^ has been widely used since its publication, particularly in clinical trials, although the criteria established have no prognostic value and the document focuses on defining only severe and moderate exacerbations (Table 4). These scientific societies believe that a specific definition for mild asthma exacerbation is not necessary, as changes in symptoms, PEF, or lung function during these episodes may fall within the normal range for that patient and reflect a transient loss of asthma control rather than the initial stage of a severe exacerbation. The SEPAR/ALAT consensus, therefore, proposes that non-severe asthma exacerbation be defined as an event that worsens the patient's baseline situation and, when recognized, requires additional treatment to prevent progression to a severe exacerbation. These exacerbations can be identified in real time or retrospectively by the presence of all the criteria established in this consensus (Fig. 1).

**Table 4:** ERS/ATS/EAACI definitions of asthma by severity.

Publication	Definition
*Severe exacerbation*	
Reddel HK et al., 2009^24^	**Severe asthma exacerbations** are defined as events that require urgent action on the part of the patient and physician to prevent a serious outcome, such as hospitalization or death from asthma. The definition should include at least one of the following: (1) Use of systemic corticosteroids or an increase from a stable maintenance dose, for at least 3 days. (2) A hospitalization or emergency department visit because of asthma, requiring systemic corticosteroids.
Chung KF et al., 2014^11^	Uncontrolled asthma is defined as at least one of the following: (1) Poor symptom control: ACQ consistently 1.5, ACT, 20 (or “not well controlled” by NAEPP/GINA guidelines) (2) Frequent **severe exacerbations**: 2 or more bursts of systemic CS (≥3 days each) in the previous year (3) **Serious exacerbations**: at least one hospitalization, ICU stay or mechanical ventilation in the previous year (4) Airflow limitation: after appropriate bronchodilator withhold FEV_1_ < 80% predicted (in the face of reduced FEV_1_/FVC defined as less than the lower limit of normal) CS, corticosteroids; ACQ: Asthma Control Questionnaire; ACT: Asthma Control Test; NAEPP National Asthma Education and Prevention Program
Bourdin A et al., 2019^14^	A definition based on 5 days of OCS is preferred to 3 to better fit with the recognized harmfulness of cumulative doses of OCS; a composite score assessing risk factors (age, comorbidities, history) would be helpful. Definition of severe exacerbation is stated as a research need.

*Moderate exacerbation*	
Reddel HK et al., 2009^24^	A **moderate asthma exacerbation** is an event that, when recognized, should result in a temporary change in treatment in an effort to prevent the exacerbation from becoming severe. It should include one or more of the following: deterioration in symptoms, deterioration in lung function, and increased rescue bronchodilator use. These features should last 2 days or more but not be severe enough to warrant systemic corticosteroid use and/or hospitalization. ER visits for asthma (e.g. for routine sick care), not requiring systematic corticosteroids, may be classified as moderate exacerbations. The magnitude of change in these outcomes will differ depending on the population studied and each individual patient's baseline variation.
Virchow JC et al., 2015^25^	**Moderate asthma exacerbation** should include ≥1 of the following criteria combined with a change in treatment: (a) Nocturnal awakening(s) due to asthma requiring SABA for 2 consecutive nights or an increase of ≥0.75 from baseline in daily symptom scores on 2 consecutive days; (b) Increase from baseline in occasions of SABA use on 2 consecutive days (minimum increase: 4 puffs/day); (c) ≥20% decrease in PEF from baseline on at least 2 consecutive mornings/evenings or ≥20% decrease in FEV_1_ from baseline and/or (d) visit to the emergency room/trial site for asthma treatment not requiring systemic corticosteroids.

Moderate exacerbation, according to the ERS/ATS, should be considered as an event that, when recognized, results in a temporary change in treatment to prevent it from progressing to a severe exacerbation.^24^, ^25^ This definition also includes one or more of the following criteria: deterioration in symptoms, deterioration in lung function, and increased rescue bronchodilator use for at least 2 days, but not severe enough to warrant the use of systemic corticosteroids or hospitalization. According to the ERS/ATS, emergency department visits for asthma (e.g. for routine medical care) do not necessarily require systemic corticosteroids, but they recognize that the magnitude of the changes in these outcomes may vary depending on the population studied and individual patient characteristics^24^ (Table 4).

In the SEPAR/ALAT consensus, we decided to distinguish clearly between severe and very severe exacerbations. A severe asthma exacerbation, according to SEPAR/ALAT, is an event that requires urgent action by the patient and physician to prevent a very severe outcome, and is identified, either in real time or retrospectively, by one or more of the established criteria (Fig. 1). ERS/ATS, on the other hand, does not specifically address very severe or potentially fatal exacerbations in its standard definition and focuses solely on severe exacerbations (Table 4).

In this consensus, very severe exacerbations are distinguished from severe exacerbations as events that require urgent action to prevent a fatal outcome, such as death from asthma (Fig. 1). The ERS/ATS definition of severe exacerbation includes events that require urgent action by the patient and physician to prevent a serious outcome, such as hospitalization, death from asthma, or progression to severe asthma. ERS/ATS argues that in clinical trials, the definition should be based mainly on the use of systemic corticosteroids (tablets, suspension or injectables) for at least 3 days (considering bursts administered 1 week apart as independent events) or on the need for hospitalization or emergency room visits due to an asthma attack treated with systemic corticosteroids (Table 4).

### Toward a comprehensive classification of exacerbations

An important feature of this proposal is that by offering a more precise classification of exacerbations, we avoid a “miscellaneous” category. The definitions of very severe, severe and non-severe exacerbations have been established with the aim of providing broad, but pragmatic and useful solutions and facilitating a clearer distinction between critical and less severe states. The criteria for very severe exacerbations are specific and relatively well accepted. However, the severe exacerbation category is challenging due to the uncertainty surrounding the interpretation of clinical parameters and vital signs. This is further evidence of the variability inherent in clinical practice, so the experience of the panelists and their knowledge of the disease has contributed greatly to defining this category. We found that the definition of non-severe exacerbation varies greatly in clinical practice, but since this category involves a less severe event, we can assume that a range of approaches may be acceptable in managing these cases. It was difficult to reach consensus on the criteria for treatment, as these can be based on subjective attitudes that vary among different clinical settings. In this respect, we must remember that the clinical practice of both the SC and the panelists can differ widely.

### Limitations of treatment-based definitions

It should be noted that the vast majority of definitions available in the literature and used to date in clinical trials and clinical practice are based primarily on the use of corticosteroids (systemic treatment for >3 days in the case of severe exacerbation). In this paper, we propose disassociating the definition of exacerbation from the need to administer a specific treatment, since the different settings in which an asthma exacerbation is managed can result in a variety of treatment plans. Nevertheless, the SC and the panel agree that this disassociation is not possible at present, since more intrinsic exacerbation markers are needed before we can establish a correct classification of exacerbations that would be so useful in standardizing asthma care and clinical trial outcomes.

### A consensus for clinical and research purposes

To our knowledge, this is the first time that these definitions have been addressed using an expert quantitative consensus methodology based on an in-depth analysis of the scientific literature. This consensus, enhanced by the knowledge and experience of numerous respiratory medicine experts in both Spain and Latin America, seeks to clarify the definition of asthma exacerbations. However, it is important to recognize the limitations inherent in this approach. The nature of the Delphi consensus means that, despite achieving broad agreement among experts, high-quality evidence on the definition and classification of asthma exacerbations may be scant. Our hope here is to lay the first foundations for defining asthma exacerbation according to severity, resulting in better, more standardized care for these patients and contributing to the design of future studies that allow us to accurately evaluate therapeutic efficacy and effectiveness (Fig. 1).

In conclusion, definitions and classifications of asthma exacerbations are essential for effective disease management and better quality of life. The ambiguity and variability of existing definitions underline the need for a clear consensus that allows health professionals to uniformly categorize asthma exacerbations according to severity. Despite the limitations of the present consensus, these definitions are expected to provide a useful framework for clinical practice and to promote standardized research in the future. Consistent criteria and less variability in interpretation are essential for improving the care of asthma and its exacerbations.

## Artificial intelligence involvement

No part of the study was generated or assisted by any artificial intelligence software or tool.

## Informed consent

Not applicable.

## Funding

This work has received funding from the Spanish Society of Pulmonology and Thoracic Surgery (SEPAR) and the Latin American Association of Thoracic Surgery (ALAT).

## Authors’ contributions

All authors participated in the conception and design of the study, the collection, analysis and interpretation of the data, the drafting of the manuscript, and its critical revision.

## Conflicts of interests

The authors of the article declare the following potential conflicts of interest:

AS has received honoraria for consultancy and support for research from GSK, AstraZeneca, Sanofi, Bristol, Novartis, Chiesi, Bayer, Roche and has received speaker honoraria from GSK, AstraZeneca, Sanofi, Elea, Novartis, and Teva.

FJAG has received honoraria for training and consultancy from AstraZeneca, GSK, Novartis, Orion, and Sanofi-Regeneron and support for professional activities from AstraZeneca, Sanofi-Regeneron, Chiesi, and Gebro.

LB has received payment or honoraria for lectures, presentations, speakers bureaus, manuscript writing or educational events from AstraZeneca, GSK, and Glenmark; Support for attending meetings and/or travel from AstraZeneca, GSK and honoraria for advisory boards from AstraZeneca and Sanofi.

MBA has received training and advisory fees from AstraZeneca, Chiesi, GSK, Sanofi-Regeneron, Vertex and Teva, and has received support for professional activities from AstraZeneca.

FCM has received fees for training and advisory activities from AstraZeneca, Chiesi, CSL-Behring, GSK, Grifols and Sanofi, and has received support for professional activities from these same companies.

CC has received fees for training activities and/or as a speaker for Astra Zeneca, Sanofi, Boeringer Ingelheim, and has received honoraria as an advisor to AstraZeneca.

PF has received training fees from AstraZeneca, GSK, and Sanofi, and has received support for professional activities from GSK and Sanofi.

GG has received advisory fees from GSK, Sanofi, Chiesi, Novartis, and has received support and scholarships for professional activities from GSK, Sanofi, Chiesi, Novartis and BI.

APG has received fees for training and advisory activities from AstraZeneca, GSK, Sanofi, FAES, Menarini, Bial and Chiesi.

VP has received speaking fees and advice from AstraZeneca, Chiesi, Gebro, GSK, Luminova-Medwell, Menarini, Sanofi and Trudell. He has received travel assistance to AstraZeneca and Chiesi meetings. He acts as a consultant for AstraZeneca, Chiesi and GSK.

IZ has received payment or honoraria for lectures, presentations, speakers’ bureaus, manuscript writing or educational events from AstaZeneca, GSK, and Sanofi/Regeneron.

JGSC has received training and advisory fees from Sanofi, Astra, GSK, Gebro and Menarini, and has received support for professional activities from Sanofi and AstraZeneca.

## Appendix A Supplementary data

The followings are the supplementary data to this article:
