# Supplementary material for: ALAT-SEPAR Consensus on the Definition and Classification of Asthma Exacerbations by Severity: A Move Toward International Standardization
Source: Open Respir Arch. 2025 Nov 5;7(4):100469. doi: 10.1016/j.opresp.2025.100469 (PMC12711655; doi:10.1016/j.opresp.2025.100469)
Supplement: Supplementary file 1 [file mmc1.docx]

**Supplementary material**

Table 1S. Percentage variation in disease activity states at baseline and at 1-year follow-up of people with RA.

| Start of treatment | Frequency (%) | One year | Frequency | Variation in the proportion (%) |
| --- | --- | --- | --- | --- |
| Low | 137(34,0%) | Remission | 83 (20,6%) | $\nabla$14,9 P.P |
| Low |  | Low | 33 (8,2%) |  |
| Low |  | Moderate | 18 (4,4%) |  |
| Low |  | High | 3 (0,7%) |  |
| Moderate | 229(57,0%) | Remission | 110 (27,4%) | $\nabla$33.2 P.P. |
| Moderate |  | Low | 39(9,7%) |  |
| Moderate |  | Moderate | 73 (18,2%) |  |
| Moderate |  | High | 7(1,1%) |  |
| High | 36(9,0%) | Remission | 21(5,2%) | $\nabla$6 P.P. |
| High |  | Low | 5 (1,2%) |  |
| High |  | Moderate | 5 (1,2%) |  |
| High |  | High | 5 (1,2%) |  |
| therapeutic success | | | | |
| active disease | 402 (100%) | Remission | 214(53,2%) | |
| Moderate-high | 265(65,9%) | Low | 44(10,9%) | |
